# Metagenomic Investigation of a Low Diversity, High Salinity Offshore Oil Reservoir

**DOI:** 10.3390/microorganisms9112266

**Published:** 2021-10-31

**Authors:** Gabrielle Scheffer, Casey R. J. Hubert, Dennis R. Enning, Sven Lahme, Jaspreet Mand, Júlia R. de Rezende

**Affiliations:** 1 Department of Biological Sciences, University of Calgary, Calgary, AB T2N 1N4, Canada; gabrielle.scheffe1@ucalgary.ca; 2 School of Natural and Environmental Sciences, Newcastle University, Newcastle upon Tyne NE1 7RU, UK; sven.a.lahme@exxonmobil.com (S.L.); j.de.rezende@hw.ac.uk (J.R.d.R.); 3 Faculty of Life Sciences and Technology, Berlin University of Applied Sciences and Technology, D-13347 Berlin, Germany; dennis.enning@bht-berlin.de; 4 Exxon Mobil Upstream Research Company, Spring, TX 77389, USA; jaspreet.mand@exxonmobil.com; 5 The Lyell Centre, Heriot-Watt University, Edinburgh EH14 4AS, UK

**Keywords:** Gulf of Mexico, marine subsurface, formation water, polyextremophiles, microbial adaptation, microbial persistence, metagenomics, halophiles, radionuclide resistance, metal acquisition

## Abstract

Oil reservoirs can represent extreme environments for microbial life due to low water availability, high salinity, high pressure and naturally occurring radionuclides. This study investigated the microbiome of saline formation water samples from a Gulf of Mexico oil reservoir. Metagenomic analysis and associated anaerobic enrichment cultures enabled investigations into metabolic potential for microbial activity and persistence in this environment given its high salinity (4.5%) and low nutrient availability. Preliminary 16S rRNA gene amplicon sequencing revealed very low microbial diversity. Accordingly, deep shotgun sequencing resulted in nine metagenome-assembled genomes (MAGs), including members of novel lineages QPJE01 (genus level) within the *Halanaerobiaceae*, and BM520 (family level) within the *Bacteroidales*. Genomes of the nine organisms included respiratory pathways such as nitrate reduction (in *Arhodomonas*, *Flexistipes*, *Geotoga* and *Marinobacter* MAGs) and thiosulfate reduction (in *Arhodomonas*, *Flexistipes* and *Geotoga* MAGs). Genomic evidence for adaptation to high salinity, withstanding radioactivity, and metal acquisition was also observed in different MAGs, possibly explaining their occurrence in this extreme habitat. Other metabolic features included the potential for quorum sensing and biofilm formation, and genes for forming endospores in some cases. Understanding the microbiomes of deep biosphere environments sheds light on the capabilities of uncultivated subsurface microorganisms and their potential roles in subsurface settings, including during oil recovery operations.

## 1. Introduction

Microorganisms are found in virtually all environments and have adapted to withstand a wide range of conditions in terms of pH, pressure, temperature, radiation, salinity, water content and nutrient availability [1,2,3,4]. These abiotic factors govern essential parameters that are known to constrain microbial life, such as the presence of a solvent (water), a source of energy (substrate) and a redox potential for respiration [2].

Oil reservoirs are considered extreme environments where organisms may require adaptations to high toxicity, low water availability, high temperature, high salinity, high pressure and exposure to radioactivity [5,6]. Nutrients and electron acceptors are limited, and may be inaccessible to planktonic microorganisms [5]. In deep oil reservoirs, dissolved oxygen or nitrate are normally absent as electron acceptors unless introduced during engineering interventions associated with oil recovery [5,7]. Iron and manganese are not normally in a bioavailable form for respiration. The most prevalent processes are therefore fermentation and methanogenesis [8]. Sulfate reduction can become relevant and problematic during secondary oil recovery when this electron acceptor is introduced in large quantities into the reservoir through the injection of seawater [5].

Deep biosphere microorganisms can be of practical interest, as these microorganisms may impact oil industry operations through souring and microbial corrosion [5,9], phenomena which are difficult to control over prolonged periods of time [10]. Other microorganisms can mitigate these negative effects, e.g., by utilizing injected nitrate as an electron acceptor. However, research into microbial processes like souring and souring control under hypersaline conditions is limited [11,12]. This study aimed to characterize the genomic potential of microorganisms from a deep biosphere hypersaline formation water in the Gulf of Mexico to better understand biological adaptations in a low nutrient, low electron acceptor, hypersaline environment. This is relevant for advancing knowledge of how microorganisms can impact subsurface oil operations, and in considering boundary conditions in extreme environments that determine the limits to microbial life [13].

## 2. Materials and Methods

### 2.1. Source of Formation Water

Water was collected from the oil-water separation system on an offshore platform producing oil and sweet gas (no H_2_S) from a moderately high temperature reservoir (52 °C) in the Gulf of Mexico. This highly saline water can be considered original formation water as the offshore asset does not use seawater injection for secondary oil recovery, i.e., no external water is injected into the reservoir. In addition, no biocides or corrosion inhibitors had been applied. A brand new 5-gallon steel drum with an internal polytetrafluoroethylene (PTFE) liner was rinsed with deionized water and then flushed with the same produced water being sampled prior to being filled to the brim with the anoxic water and capped. This sample was shipped to shore at ambient temperature and arrived in a laboratory in Texas, USA, 14 days later. Using anoxic and aseptic technique the water was transferred into sterile 2 L glass bottles under an atmosphere of 21% CO_2_ (balance N_2_) that were capped with butyl rubber stoppers. The water was then shipped to Newcastle University, UK, where it was received within three weeks and stored at 4 °C upon receipt. Within 5 days, 10 mL samples were filtered onto a sterile autoclaved 0.2 μm polycarbonate membrane filter (Whatman, UK) and frozen at −80 °C for DNA extraction.

Additional water samples for DNA extraction were obtained from two anoxic enrichments of the formation water. One enrichment was amended with acetate (5 mM), propionate (0.5 mM), butyrate (0.5 mM), bicarbonate (20 mM), nitrate (4 mM) and sulfate (3 mM); the sulfate was completely precipitated as BaSO_4_ owing to an excess of barium in the produced water. The other enrichment was left unamended, consisting only of 150 mL of the formation water in an anoxic, sealed serum bottle. Incubations were performed at 30 °C, and after 247 days a 10 mL aliquot was removed and frozen at −80 °C for DNA extraction. Both enrichments contained mild steel coupons (less than 0.002 mm/y corrosion was measured, and is not the focus of the present study).

### 2.2. Water Analysis

Formation water samples for geochemical analyses were taken during field collection and upon arrival at the Texas laboratory. Analyses were performed by Intertek (Houston, TX, USA; Supplementary Material File S1). Concentrations of anions and organic acids were measured by ion chromatography using a Dionex ICS (Thermo Scientific, Waltham, MA, USA). Concentrations of Al, Ba, B, Cd, Ca, Cr, Cu, Fe, Pb, Li, Mg, Mn, Ni, P, K, Si, Na, Sr and Zn were determined using inductively coupled plasma spectroscopy.

### 2.3. DNA Extraction, 16S rRNA Gene Sequencing and Analysis

DNA from the formation water and the two enrichment incubations was extracted using the MoBio Powersoil DNA Isolation kit (MO BIO, Carlsbad, CA, USA). A clean (unused) 0.2 μm polycarbonate membrane filter was used as a negative control for the extractions. DNA extracts were first used for 16S rRNA gene amplification (30 cycles) and sequencing on an Ion Personal Genome Machine (Ion Torrent, Life Technologies, Paisley, UK), using primers F515 and R926 [14] and procedures as described previously [15]. A fastq file was generated and further analyses were conducted with mothur [16] software, version 1.39.5. Reads were initially trimmed using trim.seqs with the following parameters: maxambig = 0, maxhomop = 6, bdiffs = 0, pdiffs = 0, minlength = 200, keepfirst = 400, flip = F. Further data processing and analyses based on operational taxonomic units (OTUs) followed guidelines available at http://mothur.org/wiki/454_SOP [17] (accessed on 1 December 2017) with minor modifications. OTUs were defined as sequences sharing 97% identity. Taxonomic assignment from mothur was further confirmed for representative sequences through the RDP Naïve Bayesian rRNA Classifier version 2.11 [18] using the RDP 16S rRNA training set No 18 (July 2020). All raw sequence data files (fastq format) generated in this study are available through the NCBI Sequence Read Archive (Bio-Project accession number PRJNA752507).

### 2.4. Metagenomics

#### 2.4.1. Metagenome Sequencing

To maximize DNA template input for shotgun metagenomic sequencing, additional aliquots of the original formation water (total 90 mL) and the enrichments (total 30 mL) were filtered. DNA was extracted as described above and combined with remaining DNA from the extracts used for amplicon sequencing. DNA amounts derived from the original formation water, the amended and the unamended enrichments were determined using a Qubit dsDNA HS Assay Kit (Life Technologies, Paisley, UK) as being 170, 35 and 44 ng, respectively. Metagenomic sequencing was carried out by NU-OMICS (Northumbria University, Newcastle upon Tyne, UK) using the Nextera XT library preparation kit following manufacturer’s instructions. Libraries were diluted to a final concentration of 8 pM and sequenced using a V3 600 cycle kit on an Illumina MiSeq platform.

#### 2.4.2. Processing of Metagenome-Assembled Genomes (MAGs)

BBDuk was used for quality control of the reads [19]. The last 151 bp of the reads and the partial adapters were trimmed off, contaminants were filtered out, low quality ends were removed and FastQC was used to evaluate sequence quality [19,20]. To increase the likelihood of resolving high quality MAGs, co-assembly was performed on the three samples used in this study. Megahit was used for co-assembly and the kmax value was set to be longer than the size of the median sample to improve quality. Bowtie2 was used for mapping the fasta files with new simple definitions to enable further processing of mapping files in Anvi’o [21,22]. Fasta files were then sorted and indexed using SAMtools [23]. For binning, Metabat was used, and MAGs 1, 3 and 7 were imported to Anvi’o for refinement [21,24]. MAGs that were at least 50% complete with less than 10% redundancy were retained for further investigation. CheckM and GTDB-Tk were used to assess MAG quality and assign taxonomy [24,25,26]. Annotation was performed using DRAM [27].

#### 2.4.3. Metabolic Predictions

Gene annotations were confirmed by verifying protein functions using the Kyoto Encyclopedia of Genes and Genomes (KEGG) [28]. To investigate salt adaptation, gene lists from Daly et al. [29] were used as a guide. DetR DB was used to look for radiation resistance genes [30]. To assess endosporulation potential, 237 genes listed in Jones et al. [31] were used as a guide. BlastP alignments of proteins with >30% identity, a bit score over 50 and an E-value lower than 1 × 10^−3^ were considered matches for MAGs 6 and 7 [32]. Information on retrieved genes from any database is available in Supplementary Material File S2.

#### 2.4.4. Phylogeny

Phylogenetic trees were constructed using CheckM version 1.1.3 [25] and included 2052 finished and 3604 draft genomes obtained from the Integrated Microbial Genomes (IMG) database. Genes were aligned under GAMMA and WAG models [25]. Nodes were interpreted as bootstrap values and the tree was built using Dendroscope for visualization [25,33].

## 3. Results and Discussion

### 3.1. Formation Water Chemistry

Common anions and cations were measured within the formation water samples. Concentrations of sulfate, nitrate, nitrite and most organic acids were below the limits of detection (Supplementary Material File S1). The acetate concentration was 7.1 mg/L (0.12 mM). The absence of measurable sulfate is likely due to the presence of barium (594 mg/L; 4.33 mM), leading to the precipitation of barite (BaSO_4_). The high salinity of this formation water is reflected by sodium and chloride concentrations being 45,404 mg/L (1.97 M) and 83,211 mg/L (2.35 M), respectively. These values are similar to those of other highly saline oil reservoirs reported in other studies [34,35,36,37]. Reservoirs in the Antrim shale formation (United States) have concentrations of 1080 to 40,365 mg/L sodium (45.04 mM to 1.68 M) and 4284 to 124,000 mg/L chloride (136.08 mM to 3.50 M) [38]. Barium, calcium, magnesium and strontium were also reported at higher concentrations than other metals in the samples at 594 mg/L (4.33 mM), 5434 mg/L (135.60 mM), 2005 mg/L (82.51 mM) and 633 mg/L (7.22 mM), respectively. These high metal concentrations remain within the range reported in other oil reservoir formation waters [39,40,41].

### 3.2. Microbial Community Composition

**Figure 1:**
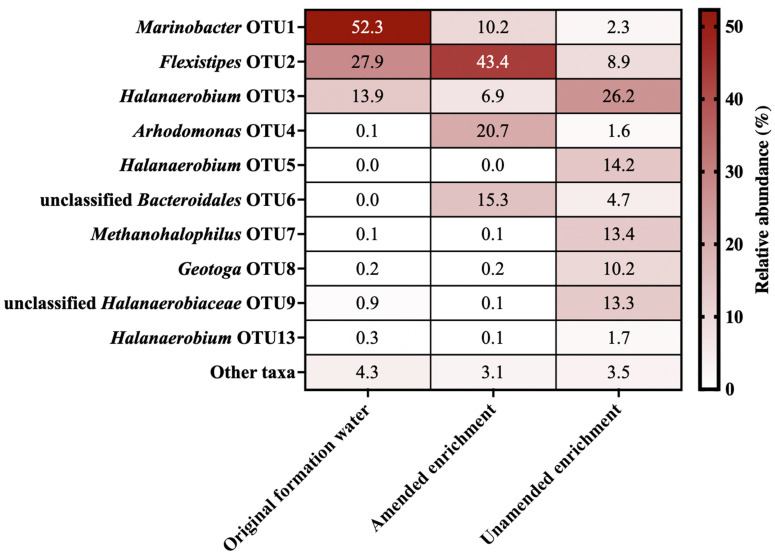
Microbial community composition in the original formation water and in amended and unamended enrichments incubated for 247 days. OTUs are defined by 97% sequence identity of 16S rRNA gene sequences. Community compositions are based on comparing 10,000 sequence reads per sample. OTUs with relative sequence abundances below 1% are grouped together as “Other taxa”. OTUs 6 and 9 have taxonomic assignments lower than 80% and could not be classified at the genus level.

Microbial diversity was first assessed by 16S rRNA gene sequencing. The community composition revealed by amplicon sequencing of the produced water and enrichment cultures is summarized in Figure 1. Surprisingly, despite amplicon libraries having over 10,000 reads, only 10 OTUs from 7 genera were identified across both the original formation water and the two enrichment cultures (based on a cut-off of 1% relative abundance; Figure 1). This unusually low diversity illustrates the extreme conditions of this high-salinity environment, and is believed to be a good reflection of the in situ diversity owing to the unlikelihood of contamination taking hold in a high salinity sample as well as the potential for DNA preservation in high salt solutions [42]. All three samples were also investigated by metagenomic sequencing that targeted genome assemblies to better understand the physiology of organisms living in the reservoir. A co-assembly strategy that also incorporated metagenomes of the two enrichments revealed genomes from organisms that were presumably present in low abundance in the original formation water, in addition to genomes corresponding to the taxa that were likely dominant in situ.

### 3.3. Metagenome Assembled Genomes and Key Metabolisms from a High Salinity Offshore Oil Reservoir

Nine metagenome-assembled genomes (MAGs), shown in Table 1, were obtained from co-assembly of the three samples. As observed in the 16S rRNA gene dataset (Figure 1), most MAGs belong to genera commonly detected in subsurface petroleum reservoir systems, including *Geotoga*, *Halanaerobium*, *Flexistipes*, *Marinobacter*, *Methanohalophilus* and *Arhodomonas* [5,29,43,44]. In addition, MAGs for two novel organisms, classified at the genus level as QPJE01 (within the family *Halanaerobiaceae*) and at the family level as BM520 (within the order *Bacteroidales*), were recovered.

Reconstructed genome completeness for the novel lineages QJPE01 and BM520 was 100% and 98.59%, respectively, with very low redundancy (Table 1). Estimated genome completeness is based on the recovery of marker genes present within the genome, and redundancy (also known as contamination) is based on the number of copies of certain marker genes as determined by both CheckM and Anvi’o [25,45]. As such, for other MAGs with lower completeness such as *Geotoga* (MAGs 1 and 9), *Methanohalophilus* (MAG 3), and *Halanaerobium* (MAG 6) it is not possible to determine whether the absence of certain metabolic features (see below) is due to the true genotype or is rather due to the lower genome completeness (Table 1). Metabolic pathways found within the MAGs are summarized in Table 2 and detailed in Supplementary Material File S2. While these organisms and genes are derived from the high salinity oil reservoir, these metagenomic findings and analyses do not necessarily reflect the structure of the in situ microbial community within the oil formation. The discussion below explores the functional capabilities of these members of the microbiome, which may be present in high relative abundance in situ or are less abundant but able to grow quickly given appropriate conditions, such as those presented in the enrichment cultures.

Succinate production potential was detected within the *Arhodomonas*, *Marinobacter*, *Halanaerobium* and *Geotoga* MAGs (Table 2). Based on the presence of acetate (7.1 mg/L; 0.12 mM) and absence of common electron acceptors in the formation water (sulfate, nitrate, carbonate; see Supplementary Material File S1), it is likely that organisms found within this environment rely mainly on fermentation. Potential for hydrogen metabolism was observed in *Flexistipes* (MAG 5) and *Geotoga* (MAG 9), which contained [NiFe]-hydrogenase genes. *Geotoga* (MAG 9) also contains *hydA*, which is essential for hydrogen production in other organisms such as *Clostridium* [46]. The final step for ethanol production is catalyzed by alcohol dehydrogenases that reduce organic compounds to ethanol, re-oxidizing NADH to NAD+ [47]. These enzymes are encoded within most of the MAGs including *Geotoga* (MAGs 1 and 9), *Arhodomonas* (MAG 2), *Marinobacter* (MAG 4) and *Halanaerobiaceae* (MAGs 6 and 7). Numerous peptidases were identified in all MAGs except the archaeal *Methanohalophilus* (MAG 3). *Arhodomonas* (MAG 2), *Marinobacter* (MAG 4), *Flexistipes* (MAG 5), QPJE01 (MAG 7) and *Geotoga* (MAG 9) genomes show potential for amino acid fermentation involving aminopeptidase and aminotransferase enzymes [29].

Both *Halanaerobiaceae* genomes (MAG 6 and 7) showed potential for solely fermentative metabolism including succinate production (MAG 6), ethanol production (both MAGs) and peptidase activity (MAG 7). Whereas *Halanaerobium congolense* MAG 6 is only a partially complete genome (59%), the absence of any other metabolic pathways within the reconstructed genome of QJPE01 (MAG 7) with 100% completeness suggests that this novel lineage is an acetoclastic, ethanol degrading organism that relies on fermentation. The type strain *H. congolense* that is closely related to MAG 6 is a fermenter that produces acetate, hydrogen and carbon dioxide [48].

#### 3.3.1. Carbon Metabolism

##### Fermentation

Several MAGs contained genes indicative of fermentative metabolism. Identified pathways include acetogenesis, succinate production (glyoxylate cycle), hydrogen production, ethanol production and peptide degradation [49]. All nine MAGs from the formation water contained genes for the Wood-Ljungdahl pathway (acetate production). 

##### Hydrocarbon Biodegradation

*Arhodomonasaquaeolei* (MAG 2) contains genes associated with the aerobic metabolism of benzene and toluene. Benzene can be converted to phenol and further converted to catechol by the phenol/toluene 2-monooxygenase (*dmpKLMNOP* and *tmoABCDE* gene clusters) [50]. The same gene clusters can also convert toluene to 2-hydroxytoluene and then to 3-methylcatechol [50]. Both catechol and methylcathecol intermediates can be further metabolized to formate or pyruvate and integrated into the glyoxylate or glycolysis pathways, respectively [29]. In MAG 2, the pathway for catechol compound conversion to pyruvate is incomplete despite this genome’s >98% estimated completeness, suggesting this organism uses the glyoxylate pathway (80% of these genes were detected in MAG 2). *Arhodomonas aquaeolei* was reported previously to be able to convert phenol to catechol [51,52,53], but these studies did not highlight benzene or toluene degradation for this species.

##### Methanogenesis

*Methanohalophilus euhalobius* (MAG 3) was the only methanogen found within the formation water sample. This organism is commonly detected in highly saline oil reservoirs and is known as an obligate methylotrophic methanogen [54]. *Methanohalophilus* spp. are predominant halophilic methylotrophic methanogens within the order *Methanosarcinales*, and use the classical methylotrophic pathway that converts methylated compounds to methane and carbon dioxide; MAG 3 has all genes associated with this pathway [54].

#### 3.3.2. Sulfur Metabolism

##### SOX System

**Figure 2:**
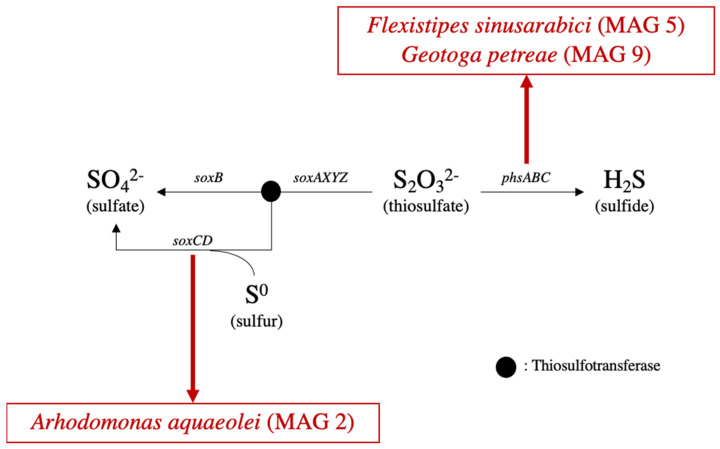
Genes present within MAGs for thiosulfate oxidation (SOX system—**left branch**) and thiosulfate reduction (**right branch**).

The SOX system in bacteria allows for thiosulfate and elemental sulfur oxidation to sulfate and is a major part of the sulfur cycle [55]. Some but not all genes that are part of the thiosulfate oxidation pathway were found in *Arhodomonas* (Figure 2). MAG 2 also includes *soxC* and *soxD* genes, which are involved in the conversion of elemental sulfur to sulfate [55]. An incomplete SOX pathway in a nearly complete genome (98.59% completeness) suggests that this *Arhodomonas* can potentially oxidize elemental sulfur, which has been reported to accumulate in some sour reservoirs [56]. Oxidation of sulfur to sulfate could potentially generate an electron acceptor for the production of sulfide through dissimilatory sulfate reduction [57].

##### Thiosulfate Reduction

Reduction of thiosulfate to sulfide can be mediated by *phsABC* (Figure 2), which were found within the genomes of *Flexistipes sinusarabici* (MAG 5) and *Geotoga petraea* (MAG 9). Sulfide production by members of both of these genera has been reported previously [43,58]. *Halanaerobium congolense* is also a thiosulfate and elemental sulfur respiring organism [48]; however, associated genes were not detected in closely related MAG 6, possibly due to its low genome completeness (59%; Table 1).

#### 3.3.3. Nitrogen Metabolism

##### Dissimilatory Nitrate Reduction to Ammonia (DNRA)

**Figure 3:**
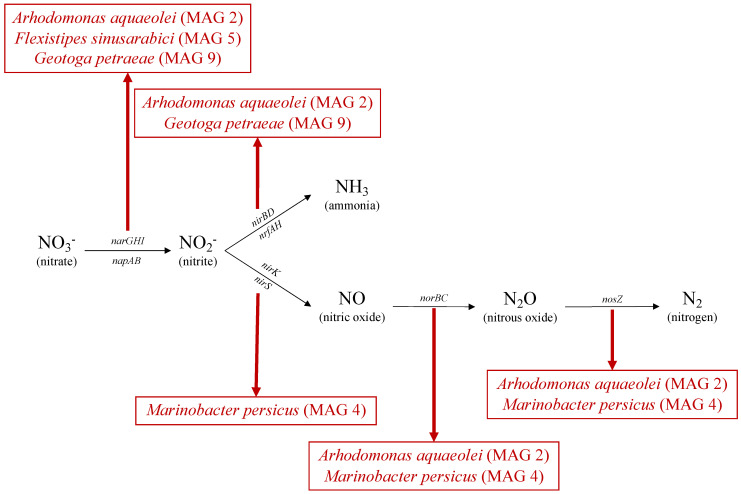
Genes present within MAGs for dissimilatory nitrate reduction (top branch) and denitrification (bottom branch).

The Nar complex consists of three different subunits (GHI) involved in the reduction of nitrate to nitrite, as shown in Figure 3 [59]. *Arhodomonas aquaeolei* (MAG 2) is known to reduce nitrate but not nitrite [44], consistent with *narGHI* genes being identified within the genome of MAG 2 (Figure 3). This Nar gene complex was also found in the genome of *Flexistipes sinusarabici* (MAG 5), contrary to another study of a different member of this species [58]. The same complex was also found within *Geotoga* (MAG 9), consistent with *Geotoga* spp. being reported to grow under nitrate-reducing conditions [60]. In this reservoir, *Geotoga petrae* (MAG 9) is shown to contain these DNRA genes. In addition to the Nar complex, *napA* (nitrate reductase cytochrome; Figure 3) was identified in *Arhodomonas* (MAG 2) and *Flexistipes* (MAG 5) genomes, as demonstrated previously in *Flexistipes sinusarabici* [58]. *Arhodomonas aquaeolei* (MAG 2) and *Geotoga petraea* (MAG 9) contain the *nirBD* and *nrfAH* genes involved in the reduction of nitrite to ammonia, the second step in DNRA (Figure 3).

##### Denitrification

For complete denitrification, *narGHI* and/or *napAB* (common to DNRA and denitrification), *nirK* or *nirS*, *norBC* and *nosZ* are typically required to reduce nitrate to dinitrogen gas, as shown in Figure 3. The *Arhodomonas* genome (MAG 2), in addition to containing genes for dissimilatory nitrate reduction (encoding *narGHI*), also encodes nitric- and nitrous oxide reductases (*norBC* and *nosZ*, respectively) that catalyze the final two steps in the denitrification pathway (Figure 3). Similar findings were observed in the *Marinobacter persicus* genome (MAG 4), where *nirS*, *norBC* and *nosZ* are present, but *narGHI* is missing. Given the high completeness of MAG 4 (92.96%), these findings suggest that nitrate may not be metabolized by this *Marinobacter*, which can still potentially reduce nitrite to nitrogen gas. Other members of the *Marinobacter* genus have been reported to be able to catalyze complete denitrification [61].

#### 3.3.4. Nitrogen and Sulfur Metabolism in the Context of Souring and Souring Control

Addition of nitrate to control sulfide-producing organisms has the potential to mitigate reservoir souring [11,12,60,62,63]. In this context, nitrate-reducing microorganisms (NRM) are able to influence sulfide production by two main mechanisms: biocompetitive exclusion of sulfide-producing organisms via NRM oxidizing electron donors that would otherwise support sulfate reduction, and nitrate reduction directly coupled to sulfide oxidation by chemolithotrophic NRM [62]. Whether these mechanisms are operative under highly saline conditions, such as those in this reservoir, has not received a lot of investigation. An et al. [11] showed that nitrite accumulation successfully controlled souring in enrichment cultures of samples from the Bakken shale formation, revealing the potential for nitrite inhibition of SRB at higher salinities. In the oil reservoir studied here, four MAGs exhibit the potential for nitrate metabolism, either via DNRA in *Flexistipes* (MAG 5) and *Geotoga* (MAG 9), or via denitrification in *Arhodomonas* (MAG 2) and *Marinobacter* (MAG 4). These MAGs likely represent organotrophic NRM, as no sulfide oxidizing genes were detected (Section 3.3.2). This suggests biocompetitive exclusion as a more likely mechanism for nitrate-based control of sulfide-producing organisms like *Flexistipes* (MAG 5) and *Geotoga* (MAG 9) if this reservoir were to sour. Overall, the in situ microbial community of this high salinity reservoir shows the metabolic potential for souring control via nitrate injection if this measure is needed during future operations.

### 3.4. Novel Lineages

#### 3.4.1. Reconstructed Genome of BM520

**Figure 4:**
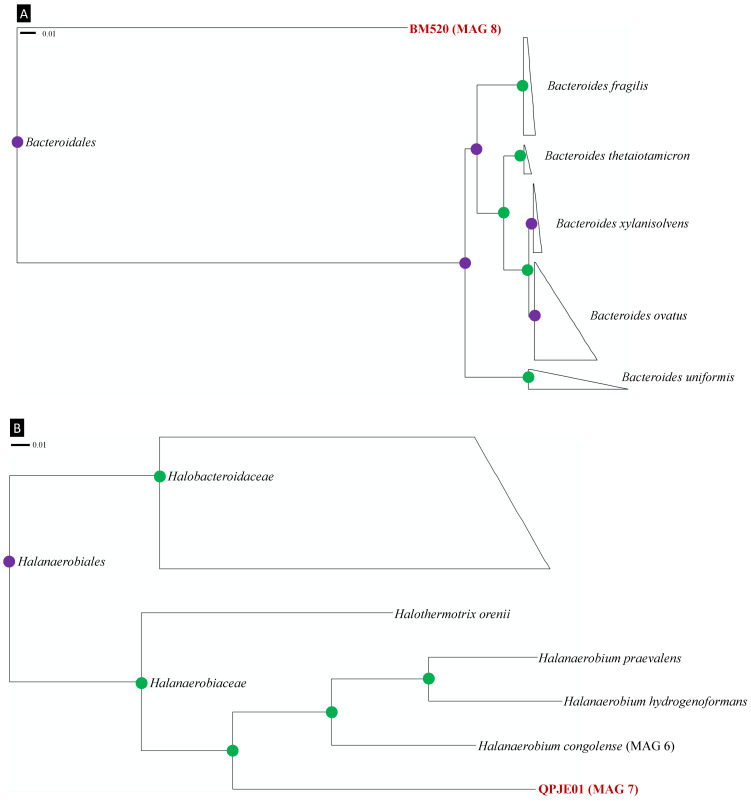
Phylogeny of novel lineages detected in oil reservoir formation water affiliated with (**A**) the order Bacteroidales, and (**B**) the family Halanaerobiaceae. The tree is based on CheckM phylogeny and only shows selected lineages from the orders Bacteroidales and Halanaerobiales. Green circles represent bootstrap values of 1.000 and purple circles represent bootstrap values between 0.900 and 0.999. Novel organisms are highlighted in bold red. The scale bars indicate 1% sequence divergence.

Members of the order Bacteroidales are commonly found in oil reservoirs [64], including those that have experienced biodegradation. This has led to speculation about their potential involvement in this process as fermentative partners for methanogens [65,66,67]. Bacteroidales have been found in onshore and offshore oil reservoirs in Canada, China, the Danish North Sea, Gabon, the Norwegian Sea and the United States [64,68]. As shown in Figure 4A, the high bootstrap support for separating the BM520 family from other Bacteroidales adds confidence to its classification as a separate group. Despite the high completeness of the BM520 genome (98.59%) and its genome size (3.5 kB), very few recognized metabolic genes were detected. Some Bacteroidales can produce acetate when degrading organic compounds in syntrophy with hydrogen producers, in agreement with the BM520 genome discovered here (Table 2) [67,69]. MAG 8 also includes genes associated with butyrate production *but* (butyrate kinase) and *ptb* (phosphate butyryltransferase) (data not shown), similar to observations for Bacteroidales in the human gut [70]. Discoveries of organisms and genomes such as BM520 will help to further elucidate the biogeochemical role of Bacteroidales in oil reservoirs.

#### 3.4.2. Reconstructed Genome of QPJE01

Members of the genus QPJE01 are distantly related to *Halanaerobium* spp., which are prevalent in oil fields, especially those produced by hydraulic fracturing (Figure 4B) [71,72]. Organisms within the family Halanaerobiaceae are often predominant in high salinity reservoirs with temperatures below 50 °C [72]. Bootstrap values throughout the Halanaerobiaceae phylogeny shown in Figure 4B support QPJE01 representing a novel genus within this family. The genome completeness (100%) and contamination (1.41%) for MAG 7 allow its metabolic potential to be inferred with greater confidence. Members of the related genus *Halanaerobium* are considered thiosulfate-reducing sulfidogens that are also capable of fermentative metabolism [71,73]. Genes associated with fermentation are abundant in QPJE01 MAG 7 (see Section 3.3.1); however, thiosulfate reduction and sulfide production genes were not found in this genome (Section 3.3.2; Table 2).

### 3.5. Adaptation to Extreme Environmental Conditions

#### 3.5.1. Salt Adaptation

Microorganisms that are able to function in highly saline environments do so by implementing either the “salt-in” or “salt-out” strategy [74]. The salt-in strategy involves the exclusion of sodium ions and accumulation of potassium ions in the cell to balance osmotic pressure with the environment [75]. Certain halophilic and fermentative organisms accumulate sodium ions rather than potassium ions for this purpose [76]. The salt-out strategy depends on the accumulation of compatible solutes (organic molecules) that maintain osmotic pressure while removing intracellular salts [29,75]. Different organic molecules can be synthesized or taken up from the surrounding environment to counter the effect of salinity. These include glycine betaine, hydroectoine, polar amino acids (e.g., proline) or sugars (e.g., sorbitol, trehalose, maltose, mannitol) [29,75]. MAGs were examined for genomic evidence for these salt adaptation strategies.

##### Salt-In Strategy

Potassium uptake systems expressed by *trkH* and *phaABCDEFG* were detected in all bacterial genomes, but not in the archaeal genome of *Methanohalophilus euhalobius* (MAG 3; 76% completeness), which included only a single sodium uptake system (*mnhABCDEFG*). A different sodium uptake system (*nhaC*) was detected in *Geotoga* (MAG 9), *Marinobacter* (MAG 4), *Halanaerobium* (MAG 6) and QJPE01 (MAG 7) genomes.

##### Salt-Out Strategy

**Figure 5:**
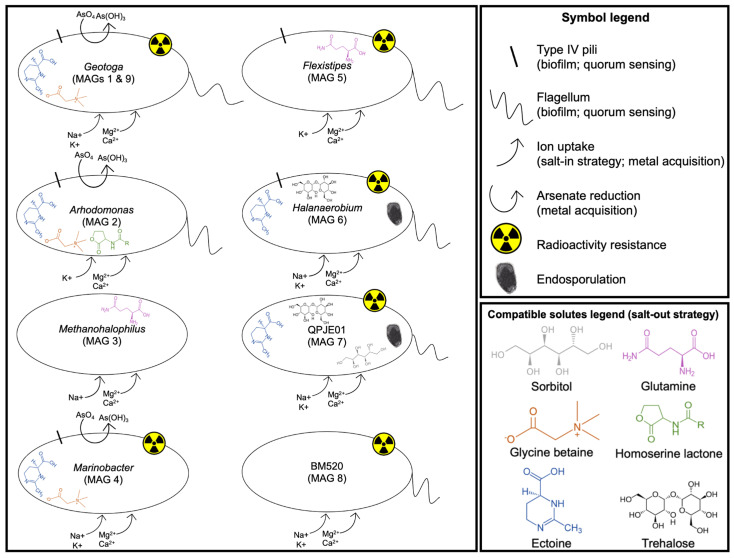
Potential environmental adaptations encoded in genomes from members of a Gulf of Mexico oil reservoir microbiome. Results from two *Geotoga* genomes (MAGs 1 and 9) are similar and are depicted together.

Ectoine synthesis genes (*ectABCD*) were found in the *Arhodomonas* (MAG 2) and *Marinobacter* (MAG 4) genomes. These two genomes also contain transporter genes for glycine betaine (*betABL*) and proline (*proPQVWX*), whereas *opuABCD* was found in *Arhodomonas* (MAG 2), and *betABC* and *opuABCD* were found in *Marinobacter* (MAG 4). Ectoine/hydroxyectoine transporter genes (*ehuABCD*) were found in *Flexistipes* (MAG 5), *Halanaerobium* (MAG 6) and QJPE01 (MAG 7) genomes. The *Flexistipes* genome (MAG 5) also contains genes for a glutamine transport system, and glycine/sarcosine methyltransferase genes were found in the genome of *Methanohalophilus* (MAG 3). Trehalose synthesis genes (*otsAB*) were only found in the two Halanaerobiaceae genomes (MAG 6 and 7), and QJPE01 (MAG 7) was the only genome that contained *srlABE* genes involved in sorbitol transport. Interestingly, no evidence of these known salt adaptation mechanisms was observed in the Bacteroidales genome (BM520; MAG 8) despite its 98.59% completeness. This is perhaps consistent with the general lack of recognizable genes within this genome (see Section 3.4.1 above), suggesting that this novel organism is adapted to high-salt environments utilizing another type of mechanism. These results are summarized in Figure 5.

#### 3.5.2. Radiation Adaptation

Oil reservoirs have been reported to have varying levels of naturally occurring radionuclides due to water-rock interactions [5]. In saline oil reservoirs with high chloride levels, the dissolution of radioactive materials may be accelerated [77]. Despite this, radiotolerant microorganisms in oil and gas reservoirs have received little attention relative to the focus placed on the non-reservoir obligate aerobe *Deinococcus radiodurans* and its bioremediation of radioactive waste [78]. The DetR DB database was created to track radioactive-resistant microorganisms and the genes that they encode that are important for radiation resistance. DetR DB reports 22 radiation-resistant organisms with three genes in common among all of them [30]. Nudix hydrolases are a protein family which hydrolyses nucleotides to regulate their level and eliminate potentially toxic derivatives [79]. SufB is important for increasing intracellular free iron, which helps repair radiation damage [80]. MutS is an endonuclease which is associated with DNA mismatch repairs [78]. These three genes were found in six out of the nine high salinity oil reservoir MAGs recovered in this study, i.e., *Marinobacter* (MAG 4), *Flexistipes* (MAG 5), *Halanaerobium* (MAG 6), QPJE01 (MAG 7), BM520 (MAG 8) and *Geotoga* (MAG 9).

#### 3.5.3. Metal Acquisition

Metal ions are necessary for organisms owing to their roles as cofactors for metabolic processes [81]. Barium and strontium have been considered as inert and non-essential to microbial life, so even though they were in higher concentrations in the sample, these metals were not considered in genomic investigations that rather focused on Mg^2+^ and Ca^2+^ and their known biological effects [82,83,84]. In addition to extremely high salinity in the oil reservoir, 5434 mg/L calcium (135.60 mM) and 2005 mg/L magnesium (82.51 mM) were detected in the formation water (Section 3.1).

All nine MAGs included genes for magnesium and calcium acquisition mechanisms. Magnesium has been reported to be toxic at around 500 mg/L (20.58 mM), while maximum intracellular calcium levels are usually kept around 4 mg/L (0.10 mM) [85]. All MAGs had at least one of the genes responsible for magnesium uptake processes (*mgtE* or *corA*), and all had *yrbG* for calcium uptake. Genes for the reduction of arsenate were detected in *Geotoga*, *Arhodomonas* and *Marinobacter* genomes (MAGs 1, 2, 4 and 9). The absence of respiratory arsenate oxidase or reductase (*asoA/B*) points to the potential for reduction of arsenate only to control its toxicity [86] and not for respiration in these organisms.

#### 3.5.4. Endosporulation

*Halanaerobium congolense* has been studied in pure culture since 1997 [48]. The genus has been detected in many oil reservoir microbial communities, usually in association with highly salinity, and has received attention in recent years in the context of hydraulic fracturing [71,87,88]. Although these organisms are persistent in oil reservoirs, only recently has it been proposed that the oil reservoir isolate *Halanaerobium congolense* has the ability to form endospores [31]. Two MAGs from the Halanaerobiaceae family (MAGs 6 and 7) were therefore assessed for the presence of genes encoding 136 endosporulation-specific proteins. For both genomes, all genes implicated in sporulation initiation (stage I), asymmetric septation (stage II), engulfment (stage III) and cortex formation (stage IV) were detected (Supplementary Material File S2). Three genes associated with the final stage of endosporulation (spore coat maturation; stage VI) are missing from both MAG 6 (*Halanaerobium*) and MAG 7 (QPJE01). Notably, mutants of *Bacillus subtilis* missing two of these genes (*spsK* and *spsL*) were still able to complete endosporulation [89]; these two genes as well as *alr* were also not found in the *H. congolense* strain reported recently as being capable of sporulation [31]. Taken together, these metagenomic results extend the potential for endosporulation more broadly among Halanaerobiaceae and within the oil reservoir microbiome.

#### 3.5.5. Quorum Sensing and Biofilm Formation

One of the ways that microorganisms act synergistically in natural or engineered environments is through biofilm formation. Quorum sensing is the ability for cell-to-cell communication for gene regulation, and is an important capability governing the establishment of multi-cellular associations such as biofilms [90]. In the context of oil and gas operations, quorum sensing and biofilm formation have both been reported as prerequisites to microbially influenced corrosion [91].

The *Arhodomonas* (MAG 2) genome has an acyl homoserine lactone synthase gene (*lasI*), which is a key component in quorum sensing regulated coordination of gene expression in a cell-density dependent manner [92,93]. Additionally, *Arhodomonas* (MAG 2), *Methanohalophilus* (MAG 3), *Marinobacter* (MAG 4), *Flexistipes* (MAG 5) and *Halanaerobium* (MAG 6) genomes have *phnAB* clusters responsible for encoding the quinolone signal, originally characterized in *Pseudomonas* and responsible for controlling gene expression [94,95]. The *Arhodomonas* genome includes a complete chemosensory system (cAMP/*vfr* signaling system) that is known to induce type II and III secretion systems and type IV pili [96]. Genes for type IV pili were detected in *Arhodomonas* and *Marinobacter* (MAGs 2 and 4), indicating potential for close associations between cells and the exchange of DNA [97]. *Geotoga* (MAG 1), *Arhodomonas* (MAG 2), *Flexistipes* (MAG 5), *Halanaerobium* (MAG 6), QJPE01 (MAG 7) and BM520 (MAG 8) genomes all contained genes for flagellar biosynthesis and assembly, which are necessary for the transition state between motility and sessility during biofilm formation. Flagella have a mechanosensory role in surface sensing and are important for the initial stages of surface adhesion that eventually lead to biofilm formation [98].

## 4. Conclusions

Formation water from a high-salinity Gulf of Mexico oil reservoir revealed a low-diversity microbial community to which a metagenomic sequencing strategy was applied to recover nine metagenome-assembled genomes with more than 50% completeness. Although the organisms investigated are most likely predominantly fermentative in this subsurface setting, potential for sulfide production, and hence reservoir souring, was detected in *Arhodomonas* and *Geotoga*. Genomic evaluation furthermore revealed the metabolic potential of this oil field microbiome to respond favorably to nitrate-based souring mitigation, with the potential for dissimilatory nitrate reduction and denitrification detected in *Arhodomonas*, *Flexistipes*, *Geotoga* and *Marinobacter*. Members of this microbiome, including two novel lineages BM520 and QJPE01, also have potential for quorum sensing and biofilm formation, and withstanding high salinity and the effects of potential radionuclide exposure in the subsurface. Endospore formation genes observed in *Halanaerobiaceae* highlight another mechanism for persistence of microbial populations in this poly-extreme environment.

## Figures and Tables

**Table 1:** Metagenome parameters and taxonomic assignments of 9 MAGs from oil reservoir formation water.

	Bin Name	MAG 1	MAG 2	MAG 3	MAG 4	MAG 5	MAG 6	MAG 7	MAG 8	MAG 9
**Sequence statistics**	**Total length (bp)**	1,452,146	3,984,249	1,369,581	2,973,006	2,998,729	2,088,230	2,003,357	3,539,121	1,490,822
	**Number of contigs**	312	327	217	100	96	227	185	151	293
	**N50**	4863	19,895	7432	52,585	79,451	12,403	15,484	33,161	5366
	**GC content (%)**	29.33	69.87	42.84	58.14	41.21	33.17	33.80	43.35	30.18
	**Completion (%)**	76.06	98.59	76.32	92.96	97.18	59.15	100.00	98.59	47.89
	**Redundancy (%)**	2.82	7.04	6.58	2.82	2.82	5.63	1.41	1.41	2.82
**Taxonomic assignation**	**Domain**	Bacteria	Bacteria	Archaea	Bacteria	Bacteria	Bacteria	Bacteria	Bacteria	Bacteria
	**Phylum**	Thermotogae	Proteobacteria	Euryarchaeota	Proteobacteria	Deferribacteres	Firmicutes	Firmicutes	Bacteroidetes	Thermotogae
	**Class**	Thermotogae	Gammaproteobacteria	Methanomicrobia	Gammaproteobacteria	Deferribacteres	Clostridia	Clostridia	Bacteroidia	Thermotogae
	**Order**	Petrotogales	Chromatiales	Methanosarcinales	Alteromonadales	Deferribacterales	Halanaerobiales	Halanaerobiales	Bacteroidales	Petrotogales
	**Family**	*Petrotogaceae*	*Ectothiodospiraceae*	*Methanosarcinaceae*	*Alteromonadaceae*	*Defferibacteraceae*	*Halanaerobiaceae*	*Halanaerobiaceae*	BM520	*Petrotogaceae*
	**Genus**	*Geotoga*	*Arhodomonas*	*Methanohalophilus*	*Marinobacter*	*Flexistipes*	*Halanaerobium*	QPJE01	-	*Geotoga*
	**Species**	*G. petraea*	*A. aquaeolei*	*M. euhalobius*	*M. persicus*	*F. sinusarabici*	*H. congolense*	sp003337245	-	*G. petreae*

**Table 2:** Summary of metabolic potential for reconstructed MAGs derived from oil reservoir formation water.

		Bin Name	MAG 1	MAG 2	MAG 3	MAG 4	MAG 5	MAG 6	MAG 7	MAG 8	MAG 9
		**Taxonomy**	*Geotoga* *petraea*	*Arhodomonas aquaeolei*	*Methanohalophilus* *euhalobius*	*Marinobacter* *persicus*	*Flexistipes* *sinusarabici*	*Halanaerobium congolense*	QPJE01	BM520	*Geotoga* *petreae*
		**Completion (%)**	76.06	98.59	76.32	92.96	97.18	59.15	100.00	98.59	47.89
**Metabolism**	**Reaction**										
Fermentation	Acetate production (Wood-Ljungdahl pathway)										
	Succinate production (glyoxylate cycle)										
	Hydrogen production										
	Ethanol production										
	Peptide degradation										
Hydrocarbondegradation	Benzene/toluene degradation										
Methanogenesis	Methylotrophy										
Sulfur metabolism	thiosulfate oxidation										
	thiosulfate reduction										
Nitrogen metabolism	Dissimilatory nitrate reduction										
	Denitrification										
Response to environment	Salt-in										
	Salt-out										
	Radiation resistance										
	Mg/Ca acquisition										
	Arsenate reduction										
	Endosporulation										
	Quorum sensing										
	Biofilm										
		Metabolic potential:									
		Present									
		Absent									
		Pathway completeness:									
		100%									
		50–99%									
		1–49%									
		0%									

## Data Availability

16S rRNA gene sequencing data and metagenomics data have been deposited at NCBI under the accession number SUB10146380 (project ID PRJNA752507).

## Supplementary Materials

The following are available online at www.mdpi.com/article/10.3390/microorganisms9112266/s1, The data in this study regarding water chemistry and metagenomic analyses are available in Supplementary Material File S1 and File S2, respectively.
