# Supplementary material for: Metagenomic Investigation of a Low Diversity, High Salinity Offshore Oil Reservoir
Source: Microorganisms. 2021 Oct 31;9(11):2266. doi: 10.3390/microorganisms9112266 (PMC8621343; doi:10.3390/microorganisms9112266)
Supplement: Supplementary file 1 [file microorganisms-09-02266-s001.zip › GabrielleScheffer_Suppl. mat. S1.pdf]

Table S1: Cation and anion concentrations from the formation water from the Gulf of Mexico oil reservoir.

Cations			Test Method	(mg/Kg)	MW	Valence	Factor	Meq/Kg
1	Aluminium	Al^{3+}	ICP	**< 0.1**	27.0	3	8.99	n/a
2	Ammonium	NH_4^+	IC	**NT**	18.0	1	18.04	n/a
3	Barium	Ba^{2+}	ICP	**594.1**	137.3	2	68.67	8.7
4	Boron	B^{3+}	ICP	**14.3**	10.8	3	3.60	4.0
5	Cadmium	Cd^{2+}	ICP	**< 0.1**	112.4	2	56.20	n/a
6	Calcium	Ca^{2+}	ICP	**5434.0**	40.1	2	20.04	271.2
7	Copper	Cu^{2+}	ICP	**< 0.1**	63.5	2	31.77	n/a
8	Chromium	Cr^{3+}	ICP	**< 0.1**	52.0	3	17.33	n/a
9	Iron (total)	Fe^{2+}	ICP	**18.8**	55.8	2	27.92	0.7
10	Lead	Pb^{2+}	ICP	**< 0.1**	207.2	2	103.60	n/a
11	Lithium	Li^+	ICP	**3.0**	6.9	1	6.94	0.4
12	Magnesium	Mg^{2+}	ICP	**2005.0**	24.3	2	12.15	165.0
13	Manganese	Mn^{2+}	ICP	**1.4**	54.9	2	27.47	0.1
14	Nickel	Ni^{2+}	ICP	**< 0.1**	58.7	2	29.35	n/a
15	Phosphorus	P^{3+}	ICP	**< 0.1**	31.0	3	10.32	n/a
16	Potassium	K^+	ICP	**332.6**	39.1	1	39.10	8.5
17	Silicon	Si^{4+}	ICP	**11.6**	28.1	4	7.02	1.7
18	Sodium	Na^+	ICP	**45404.0**	23.0	1	22.99	1975.0
19	Strontium	Sr^{2+}	ICP	**632.8**	87.6	2	43.81	14.4
20	Zinc	Zn^{2+}	ICP	**0.6**	65.4	2	32.70	0.0

Anions			Test Method	(mg/Kg)	MW	Valence	Factor	Meq/Kg
1	Acetate	CH_3COO^-	IC	**7.1**	59.04	1	59.04	0.1
2	Bicarbonate	HCO_3^-	IC	**60.1**	61.02	1	61.02	1.0
3	Bromide	Br^-	IC	**214.0**	79.90	1	79.90	2.7
4	Butyrate	$CH3CH_3CH_2CO_2^-$	IC	**< 0.9**	88.11	1	88.11	n/a
5	Carbonate	$CO_3^{=}$	IC	**0.0**	60.02	2	30.01	0.0
6	Chloride	Cl^-	IC	**83211.0**	35.50	1	35.50	2344.0
7	Fluoride	F^-	IC	**< 0.1**	19.00	1	19.00	n/a
8	Formate	$HCOO^-$	IC	**< 1.0**	45.02	1	45.02	n/a
9	Glycolate	$OHCCOO^-$	IC	**< 0.9**	76.05	1	76.05	n/a
10	Hydroxide	OH^-	IC	**0.0**	17.00	1	17.00	0.0
11	Nitrate	NO_3^-	IC	**< 0.8**	62.01	1	62.01	n/a
12	Nitrite	NO_2^-	IC	**< 1.1**	46.01	1	46.01	n/a
13	Phosphate	PO_4^{3-}	IC	**< 1.3**	94.97	3	31.66	n/a
14	Propionate	$CH_3CH_2CO_2^-$	IC	**< 0.9**	73.07	1	73.07	n/a
15	Sulphate	SO_4^{2-}	IC	**< 0.6**	96.06	2	48.03	n/a
